# A physical map for the *Amborella trichopoda *genome sheds light on the evolution of angiosperm genome structure

**DOI:** 10.1186/gb-2011-12-5-r48

**Published:** 2011-05-27

**Authors:** Andrea Zuccolo, John E Bowers, James C Estill, Zhiyong Xiong, Meizhong Luo, Aswathy Sebastian, José Luis Goicoechea, Kristi Collura, Yeisoo Yu, Yuannian Jiao, Jill Duarte, Haibao Tang, Saravanaraj Ayyampalayam, Steve Rounsley, Dave Kudrna, Andrew H Paterson, J Chris Pires, Andre Chanderbali, Douglas E Soltis, Srikar Chamala, Brad Barbazuk, Pamela S Soltis, Victor A Albert, Hong Ma, Dina Mandoli, Jody Banks, John E Carlson, Jeffrey Tomkins, Claude W dePamphilis, Rod A Wing, Jim Leebens-Mack

**Affiliations:** 1 Arizona Genomics Institute, School of Plant Sciences and BIO5 Institute for Collaborative Research, University of Arizona, 1657 East Helen Street, Tucson, AZ 85721, USA; 2 Department of Plant Biology, University of Georgia, 4504 Miller Plant Sciences, Athens, GA 30602, USA; 3 Department of Biological Sciences, University of Missouri, 371B Life Sciences Center, Columbia, MO 65211, USA; 4 College of Life Sciences and Technology, Huazhong Agricultural University, Wuhan, Hubei 430070, China; 5 Intercollege Graduate Degree Program in Plant Biology and Institute of Molecular Evolutionary Genetics, Huck Institutes of the Life Sciences, The Pennsylvania State University, 405 Life Sciences Building, University Park, Pennsylvania 16802, USA; 6 Department of Plant and Microbiology, College of Natural Resources, University of California, 311 Koshland Hall, Berkeley 94709, CA, USA; 7 Plant Genome Mapping Laboratory, University of Georgia, 111 Riverbend Road, Athens, GA 30605, USA; 8 School of Plant Sciences and BIO5, University of Arizona, 1657 East Helen Street, Tucson, AZ 85721, USA; 9 Dow Agrosciences LLC, 9330 Zionsville Road, Indianapolis, IN 46268, USA; 10 Department of Biology, University of Florida, 220 Bartram Hall, Gainesville, FL 32611, USA; 11 Florida Museum of Natural History, Museum Road and Newell Drive, University of Florida, Gainesville, FL 32611, USA; 12 Department of Biological Sciences, University at Buffalo (SUNY), 637 Hochstetter Hall, Buffalo, NY 14260, USA; 13 State Key Laboratory of Genetic Engineering, School of Life Sciences, Institute of Plant Biology, Center for Evolutionary Biology, and Institutes of Biomedical Sciences, Fudan University, 220 Handan Road, Shanghai 200433, China; 14 Northern Lights, 4500 NE 40th Street, Seattle WA 98105, USA; 15 Department of Botany and Plant Pathology, Purdue University, B028 Whistler Hall, West Lafayette, IN 47906, USA; 16 School of Forest Resources, The Pennsylvania State University, 323 Forest Resources Building, University Park, PA 16802, USA; 17 Clemson University Genomics Institute, Clemson University, 51 Cherry St, Clemson, NC 29634, USA

## Abstract

**Background:**

Recent phylogenetic analyses have identified *Amborella trichopoda*, an understory tree species endemic to the forests of New Caledonia, as sister to a clade including all other known flowering plant species. The *Amborella *genome is a unique reference for understanding the evolution of angiosperm genomes because it can serve as an outgroup to root comparative analyses. A physical map, BAC end sequences and sample shotgun sequences provide a first view of the 870 Mbp *Amborella *genome.

**Results:**

Analysis of *Amborella *BAC ends sequenced from each contig suggests that the density of long terminal repeat retrotransposons is negatively correlated with that of protein coding genes. Syntenic, presumably ancestral, gene blocks were identified in comparisons of the *Amborella *BAC contigs and the sequenced *Arabidopsis thaliana*, *Populus trichocarpa*, *Vitis vinifera *and *Oryza sativa *genomes. Parsimony mapping of the loss of synteny corroborates previous analyses suggesting that the rate of structural change has been more rapid on lineages leading to *Arabidopsis *and *Oryza *compared with lineages leading to *Populus *and *Vitis*. The gamma paleohexiploidy event identified in the *Arabidopsis*, *Populus *and *Vitis *genomes is shown to have occurred after the divergence of all other known angiosperms from the lineage leading to *Amborella*.

**Conclusions:**

When placed in the context of a physical map, BAC end sequences representing just 5.4% of the *Amborella *genome have facilitated reconstruction of gene blocks that existed in the last common ancestor of all flowering plants. The *Amborella *genome is an invaluable reference for inferences concerning the ancestral angiosperm and subsequent genome evolution.

## Background

The origin and rapid diversification of the angiosperms (flowering plants) were pivotal events in the evolutionary history of Earth's biota. Over the past 130 to 150 million years angiosperms have diversified to include approximately 350,000 species occupying nearly all habitable terrestrial and many aquatic environments. Angiosperms generate the vast majority of human food either directly or indirectly as animal feed, and they account for a huge proportion of land-based photosynthesis and carbon sequestration. Comparative analyses of genome sequences and gene function for a growing number of species are shedding light on how gene and genome duplications have contributed to the diversification within major flowering plant lineages (for example, Rosidae, Asteridae, Monocotyledoneae [1]), but elucidation of the genetic and genomic processes underlying the key innovations associated with the origin of flowering plants (for example, typically bisexual flowers, endosperm formation, double fertilization, ovules with two integuments, seed development within the carpel) requires comparisons between lineages that diverged from the last common ancestor of all extant angiosperms [2,3].

Recent phylogenetic analyses have identified *Amborella trichopoda*, an understory tree or shrub species endemic to the forests of New Caledonia, as the sister species to all other extant angiosperms [4-8]. *Amborella *is no more 'ancient' or 'primitive' than any other extant flowering plant species, but comparisons between *Amborella *and other angiosperms are allowing researchers to triangulate on characteristics of their last common ancestor. Using a similar approach, researchers have used the complete genome sequence of platypus, *Ornithorhynchus anatinus*, representing the sister group of all other extant mammals, to elucidate mammalian genome evolution [9].

Previous comparisons of transcriptome content [10], gene expression patterns [11-13], and gene function [14,15] between *Amborella *and other flowering plant species have suggested that much of the floral development program that has been characterized in *Arabidopsis*, snapdragon and maize existed in the last common ancestor of extant angiosperms. While gene duplications in the MADS-box transcription factor family likely contributed to the earliest floral development regulatory networks [11,12,16-19], it is not clear whether these were single gene duplications or the product of polyploidization. Genome duplications have occurred repeatedly throughout angiosperm history [20-23] but there is uncertainty in the timing of polyploidy events relative to the origin of the angiosperms and important innovations in flowering plant history [24].

Here we describe a BAC-based draft physical map for *A. trichopoda *and use BAC end sequences (BESs) to compare the structure of the *Amborella *genome to representative eudicot (*Vitis*, *Populus *and *Arabidopsis*) and grass (*Oryza*) genomes. Comparative analyses of sequences for two large contiguous regions (487.3 and 629.7 kb in the *Amborella *genome) were also performed. In addition we use a large transcriptome assembly to identify BAC ends matching protein-coding sequences [25]. Our aim here is to begin to investigate whether regions of these genomes have remained syntenic throughout angiosperm history, and determine whether ancient genome duplications discovered in eudicot and grass genomes [26-29] occurred before or after the divergence of these lineages from the *Amborella *lineage. In addition, the physical map and sequence analyses establish a framework for future studies of all flowering plant genomes, including the *Amborella *genome itself.

## Results and discussion

### BAC library and physical map

The structure and composition of the 870 Mbp/C [30]*A. trichopoda *genome was investigated through physical mapping of clones from a 5.2 × coverage BAC library. The library was constructed after partial digest of high-molecular-weight DNA with *Hin*dIII. The library, which comprises 36,684 BAC clones with an estimated average insert size of 123 kb, is available through the Arizona Genomics Institute [31]. The BAC library was double spotted in high density onto Hybond N+ filters. All 36,684 clones were end-sequenced, and a physical map was constructed after high information content fingerprinting (HICF) [32,33]. A total of 32,719 fingerprinted BACs was assembled into 3,106 contigs and 1,356 singletons using the program FPC version 7.2 [34].

The quality of the physical map was assessed by screening the arrayed library with probes developed for *Amborella *homologs for eight genes that have been found to be single-copy in sequenced plant genomes [35,36]. Probes derived from *Amborella *cDNA clones or PCR amplicons were putative homologs of following single-copy *Arabidopsis *genes: *ASD *(At1g14810), *DWARF1 *(At3g19820), *GIGANTEA *(At1g22770), *LEAFY *(At5g61850), a dienelactone hydrolase gene (At2g32520), a cytochrome-C-oxidase-related gene (At4g37830), *EIF3K *(At4g33250) and a hypothetical protein-coding gene with strong similarity to rice gene Os02g0593400 (At5g63135). All verified positive clones mapped to the same FPC contig for six of the eight probes (Figure S1 in Additional file 1). Positive clones for the *EIF3K *and the hypothetical protein-coding gene probes were each distributed between two FPC contigs and inspection of the HICF bands for these contigs suggests that the genes have been duplicated in the *Amborella *lineage. In accordance with the expected library coverage, the single copy nuclear gene probes hybridized to 3 to 13 clones (mean 6.9).

The correlation between HICF bands and the number of BACs included in each FPC contig was 0.655 for all contigs and 0.917 after removing two contigs derived from the chloroplast and mitochondrial genomes and one contig composed largely of repetitive elements (Figure S2 in Additional file 1). We used a calibration of average insert size (123 kb) over the average number of HICF bands per BAC clone (128) to obtain a rough estimate of FPC contig lengths. Of 77 FPC contigs with 39 or more BACs (not including the contigs with the plastome and repetitive elements), estimated lengths ranged from 308 to 1,429 kb.

BAC end sequencing was performed on all fingerprinted BACs producing 69,466 Sanger reads with an average length of 695 bp after quality and vector trimming. This corresponds to 48.25 Mbp, or roughly 5.4% of the *Amborella *genome. BESs were related to the physical map and used to identify regions of synteny between regions of the *Amborella *genome and the sequenced *Arabidopsis*, *Populus*, *Vitis *(grape), and *Oryza *(rice) genomes (see below). In addition, end sequences were used to verify the identity of the three excluded FPC contigs described above. All BESs mapping at least 100 bp apart on the plastid genome [37] were found in the same FPC contig. This contig included just 532 BACs, indicating very low (1.6%) plastid DNA contamination.

### Characterization of repeats in BAC end and shotgun sequences

Repeat composition and frequency in the *Amborella *genome were characterized through analysis of the BAC end and whole genome survey sequences. Reads were first compared with sequences in Repbase (v.15.08) [38] using BLASTN [39]. In order to minimize the effect of divergence between *Amborella *genes and homologous repeats from other species, we used relaxed BLASTN settings (-q -4 -r 5) to accommodate an estimated 160 million years of sequence divergence since the last common ancestor of extant flowering plants [8,40-42] while maintaining rigorous support for significant hits (E-value threshold was set at 1e-10). All BAC end sequences without significant hits were then compared with the non-redundant protein database in GenBank using BLASTX and an E-value threshold of e-5. Finally, the remaining sequences without matches in Repbase or the GenBank nr database were compared with sequences that did have matches in either database using BLASTN with an E-value threshold of 1.0e-10. We report results both excluding these 'internal' BLAST searches and including them (I). Together these results provide estimates of transposable element (TE) content based on conservative and more comprehensive (and possibly more permissive; I) search strategies.

With the more comprehensive strategy (I), slightly more than half of all the *Amborella *BESs matched known TE sequences. Not surprisingly, the most highly represented TE class was long terminal repeat (LTR) retrotransposons, accounting for 7.65% (I: 30.01%) of all BESs and 57.5% (I: 56.58%) of all those with hits to Repbase. Hits to Ty1-*copia *type sequences were slightly more common (3.11%; I: 13.79%) than matches to Ty3-*gypsy*-like LTRs (3.50%; I: 12.09%); the remaining LTR retrotransposon matches (1.04%; I: 4.13%) were not classified. LINEs also represented a significant fraction of *Amborella *BAC ends: 2.70% (I: 11.60%) of the total, 19.98% of all the repeats (I: 22.22%). This is noteworthy because LINEs are usually significantly less numerous than LTR retrotransposons in plant genomes [43-47] with some notable exceptions, such as the element *del2 *in *Lilium speciosum *[48]. The complete set of DNA TE-related BESs accounts for just 1.63% (I: 4.51%) of the total, and the most represented classes are those of hAT and MuDR elements: 0.92% (I: 2.41%) and 0.49% (I: 1.04%) of the total BESs, respectively. Results from the same analyses replicated on the set of 2,695 random sheared Sanger sequences (Table 1) and 648,519 454 reads (Table S1 in Additional file 1) are generally in very good agreement with those obtained using BES data.

**Table 1:** Frequencies of BAC end sequences and Sanger shot gun sequences matching sequences in Repbase

Type	Absolute number in BESs	Percentage BESs	Percentage repeats in BESs	Absolute number in SGSs	Percentage SGSs	Percentage repeats in SGSs
**DNA TEs**						
hAT	642 (1,671)	0.92 (2.41)	6.84 (4.61)	20 (41)	0.74 (1.52)	5.73 (2.94)
MuDR	343 (724)	0.49 (1.04)	3.65 (2.00)	7 (30)	0.26 (1.11)	2.00 (2.15)
CACTA	27 (75)	0.04 (0.11)	0.29 (0.21)	0 (4)	0 (0.15)	0 (0.29)
Helitrons	12 (69)	0.02 (0.10)	0.13 (0.19)	0 (3)	0 (0.11)	0 (0.22)
Other	108 (595)	0.15 (0.86)	1.15 (1.64)	1 (24)	0.04 (0.89)	0.29 (1.72)
Total	1,132 (3,134)	1.63 (4.51)	12.06 (8.64)	28 (102)	1.04 (3.78)	8.02 (7.31)
						
**Retrotransposons**						
LTR Ty1-*copia*	2,162 (9,578)	3.11 (13.79)	23.02 (26.42)	64 (314)	2.37 (11.65)	18.34 (22.51)
LTR Ty3-*gypsy*	2,431 (8,395)	3.50 (12.09)	25.89 (23.15)	129 (377)	4.78 (13.98)	36.96 (27.03)
LTR not classified	720 (2,868)	1.04 (4.13)	7.67 (7.91)	51 (139)	1.89 (5.16)	14.61 (0.96)
LINEs	1,876 (8,055)	2.70 (11.60)	19.98 (22.22)	55 (294)	2.04 (10.91)	15.76 (21.08)
SINEs	11 (183)	0.02 (0.26)	0.12 (0.50)	0 (4)	0 (0.15)	0 (0.29)
Retro not classified	1,058 (4,046)	1.52 (5.82)	11.27 (11.16)	23 (165)	0.85 (6.12)	6.59 (11.83)
Total	8,258 (33,125)	11.89 (47.69)	87.94 (91.36)	321 (1,293)	11.91 (47.96)	91.98 (92.69)
						
**Total**	9,390 (36,259)	13.52 (52.20)	100 (100)	349 (1,395)	12.95 (51.74)	100 (100)

Results in parentheses include Internal BlastN searches. Repbase v.15.08 was used [38]. SINE, short interspersed element; SGS, Sanger shot gun sequence.

A *de novo *search for novel miniature inverted repeat transposable elements (MITEs) overlooked by the similarity search approach was carried out using the pipeline MUST [49]. The most abundant candidates identified by the pipeline were manually inspected to confirm features typical of MITEs, such as small size, terminal inverted repeats, high A+T nucleotide content and target site duplications. Three putative high-copy MITEs were identified. All of these were small elements (174 to 500 bp) with terminal inverted repeats, target site duplications, and A+T content greater than 65% (Figure S3 in Additional file 1). Repeat copy numbers estimated from the BESs and random sheared sequences were extrapolated to obtain genome-wide estimates using the procedure developed by Hawkins *et al*. [50]. Copy number ranges from 3,300 copies for MITE_2 to 17,000 copies for MITE_1. The estimates inferred from BESs were generally consistent with those calculated for random sheared reads (with the possible exception of MITE_3; Table 2).

**Table 2:** Putatively high-copy MITEs identified in the BESs and Sanger shot gun sequences using MUST pipeline

	Length	Inverted repeat length	BES hits	Copy number estimate	SGS hits	Copy number estimate	AT%
MITE_1	358	26	542	~17,000	18	~17,200	68.80
MITE_2	190	19	140	~3,300	8	~3,100	68.70
MITE_3	516	47	394	~17,900	8	~11,300	75.20

Copy number estimates based on procedure of Hawkins *et al*. [50]. SGS, Sanger shot gun sequence.

**Figure 1:**
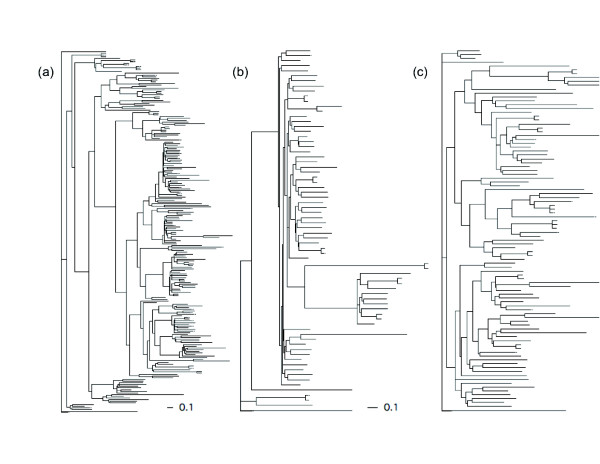
**Maximum likelihood trees for reverse transcriptase genes classified as Copia-type and Gypsy-type LTR and LINE elements**. **(a) **Copia-type; **(b) **Gypsy-type LTRs; **(c) **Gypsy-type LINEs. The maximum likelihood trees show rate heterogeneity and no recent expansive radiations (that is, short terminal branches). Reverse transcriptase sequences were mined from BAC end sequence set.

The conserved reverse transcriptase domains of LTR retrotransposons and LINEs were collected and used to estimate maximum likelihood trees (Figure 1). In the case of LTR retroelements, the trees indicate substitution rate heterogeneity (that is, variation in root-to-tip distances) and no evidence for recent retrotranspositional bursts of single families (that is, short terminal branches). In the case of LINEs, the phylogenetic tree displays very long branches suggestive of an ancient diversification or very rapid substitution rates. As has been described for other plants [51], *Amborella *LINEs exhibit high sequence divergence and extreme heterogeneity.

The *Amborella *BESs were also searched for microsatellites (that is, simple sequence repeats (SSRs)); for comparison, the search was also conducted on the *Amborella *random sheared reads and on BESs (from other *Hin*dIII BAC libraries) from *Glycine *(soybean) and *Oryza rufipogon*. In comparison to the other two species, *Amborella *shows a higher frequency of SSRs, particularly mono- and dinucleotide repeats, with a particularly high frequency of 'AG' dinucleotide microsatellites. The results of SSR analysis in BESs were confirmed by those obtained from the randomly sheared *Amborella *sequences (Table 3).

**Table 3:** Simple sequence repeats identified in BESs and Sanger shot gun sequences

Repeat	***Amborella *(BES)**^**a**^	***Amborella *(RS)**^**a**^	**SoyBean**^**a**^	***Oryza *rufipogon**^**a**^
Mono	149.66	152.89	72.74	50.79
Di	225.03	211.00	77.89	63.94
Tri	72.49	78.96	110.01	144.06
Tetra	89.88	90.70	100.67	102.25
Penta	74.85	89.73	64.54	56.00
Total	611.92	623.28	425.85	417.04

^a^Values are presence per million base pairs. RS, Random sheared

**Figure 2:**
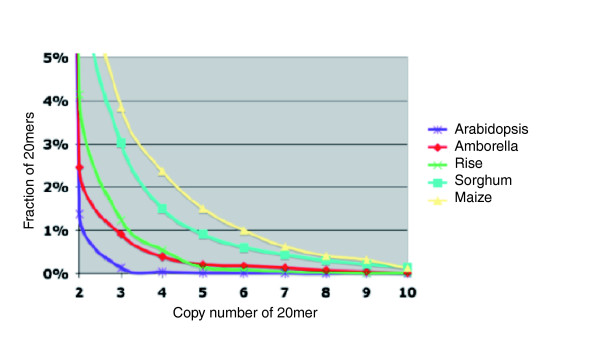
**K-mer analyses of Sanger shotgun sequences reveal low frequencies of short repeats in the *Amborella *genome relative to the sorghum and maize genomes**.

Repeat profiles in the shotgun sequences were also assessed using Tallymer to characterize K-mer frequencies [52]. The *Amborella *K-mer frequency profiles were compared with those of *Arabidopsis thaliana*, *Oryza sativa *(rice), *Sorghum bicolor *and *Zea mays *(maize). While the *Amborella *genome size is closest to Sorghum's (870 and 740 Mbp/C, respectively), its K-mer frequency profiles were more similar to those of *Arabidopsis *and rice, with much smaller genome sizes (157 and 490 Mbp/1C, respectively [53]) (Figure 2).

### Distribution of BESs with matches to protein-coding regions of reference genomes

All BESs and shotgun sequences were compared to the GenBank nr database using BLASTX [39] with an e-value threshold of 1e-5. After the removal of sequences similar to TEs, the overall frequencies of sequences finding matches in the protein database were 11.9% and 8.05% for the BES and Sanger shotgun sequences, respectively. For BESs from FPC contigs with ten or more BACs, we found a negative correlation between the frequencies of BESs matching protein-coding genes and LTR retrotransposons (r = -0.423, *P *< 0.0001). As has been described for other genomes [54-56], gene density seems to be negatively correlated with retrotranposon density in the *Amborella *genome.

### Identification of syntenic blocks between *Amborella*, *Arabidopsis*, rice, poplar and grape

Taking advantage of the availability of a phase I physical map assembly, we mapped the *Amborella *contigs onto the genomes of *A. thaliana*, *Populus trichocarpa*, *Vitis vinifera*, and *O. sativa*. We focused on the 77 largest contigs with at least 39 clones. BLAST analyses of BESs were done within the context of their linkages within FPC contigs. All of the contig BESs classified as repeats (see above) were discarded. Those remaining were compared against the four reference genomes. Because of the large evolutionary time that separates *Amborella *from the other four sequenced genomes [41,42,57], the comparisons were carried out at the protein level using tBLASTX; only the best hits were taken into account. *Amborella *FPC contigs were considered for further analyses if at least two BESs had matches with bit scores greater than 80 (typically a maximum e-value of 1.0E-20 over 100 amino acidic residues) to loci separated by less than 500 kb within one of the four genomes being compared. Positive matches were used as anchors to circumscribe 4-Mbp tracts within the reference genomes and a second, more focused tBLASTX search was performed comparing the BESs with these regions. An e-value threshold of 1.0E-4 was used for the second set of tBLASTX searches and all significant hits were used to identify syntenic regions. We considered a contig as anchored if the contig had at least four positive hits (e-value lower than 1.0e-4) to at least three distinct genes.

Non-repetitive BESs were also compared to a database of 246,196 *Amborella *cDNA unigene assemblies with lengths greater than 100 bp. These cDNAs were derived from comprehensive sequencing of nine cDNA libraries (Table 4) [25]. Sixty-six percent of the non-repetitive BESs matched cDNA sequences in BLASTN searches with an e-value cutoff of 1.0e-10.

**Table 4:** Statistics for cDNA sequences included in multi-library transcriptome assembly of 246,196 unigenes with lengths greater than 100 bp

Tissue - library name	Sequencing method	Number of reads	Unscreened reads	Total passing bases (MB)
Apical meristem - Atr12	454 FLX Titanium	794,746	688,305	201.90
Male flowers - Atr15	454 FLX Titanium	277,023	255,213	73.49
Old leaves - Atr14	454 FLX Titanium	280,097	260,563	73.49
Old stem - Atr13	454 FLX Titanium	259,431	238,156	68.70
Pre-meiotic female flower buds - Atr10	454 FLX GS	895,000	812,325	176.97
Pre-meiotic female flower bud - Atr02	Sanger	13,263	13,141	7.17
Pre-meiotic male flower bud - Atr01	Sanger	25,343	25,006	14.17
Root - Atr11	454 FLX GS	324,070	300,275	64.88
Stem - Atr16	454 FLX Titanium	410,098	388,436	120.03

Assemblies and raw data can be downloaded from the Ancestral Angiosperm Genome Project website [25]. A BLAST portal for the assembly is also available at the project website.

**Figure 3:**
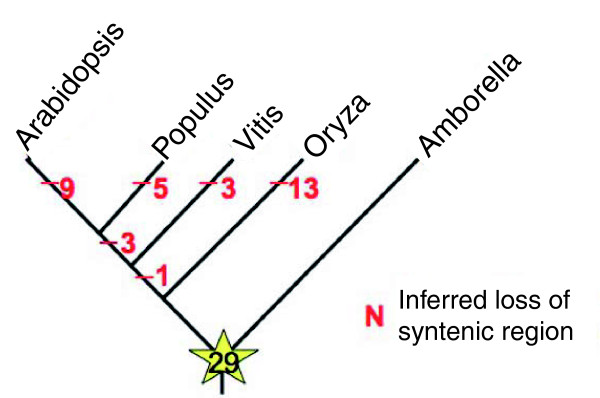
**Variation in rates of structural evolution evident in parsimony mapping of losses of synteny with 29 gene blocks inferred for the last common ancestor of all extant flowering plant lineages**.

Using the search strategy described above, 29 large *Amborella *BAC contigs (>39 BAC clones) showed synteny with at least one of the four sequenced genomes, and nine of these showed synteny with at least one region in all four genomes. All BESs mapping to these syntenic regions also exhibited significant matches to the sequences in the *Amborella *cDNA assembly (Table 4; Table S2 in Additional file 1). Whereas 25 of these *Amborella *BAC contigs mapped to at least one tract in the *Vitis *genome, 15, 16, and 24 contigs were found to be syntenic with one or more tracts in the *Oryza*, *Arabidopsis*, and *Populus *genomes, respectively (Table S2 in Additional file 1). These results provide a novel, albeit coarse, first view of the ancestral genome for all flowering plants and the timing of rearrangements and other structural changes (for example, genome duplications, fractionation, chromosomal fissions and fusions) that have reduced synteny between the monocot and eudicot genomes analyzed here (Figure 3). Parsimony mapping of synteny loss onto a phylogeny consisting of *Amborella *and the other four species indicates variation in rates of change in genome structure. In agreement with previous studies [29,45], *Vitis *seems to have been the most stable of the sequenced genomes, and the rate of change slowed in the lineage leading to *Populus *following divergence from the lineage leading to *Arabidopsis *(Figure 3).

### Paleopolyploidy in angiosperm genomes

Paleopolyploidy events have been well characterized in all four sequenced genomes analyzed here [29,45,58-60], and the syntenic *Amborella *FPC contigs described above often match multiple regions in these genomes. The most ancient of these paleopolyploidy events is the so-called γ triplication that has been inferred to have occurred before the divergence of the Asteridae (represented by tomato, *Solanum lycopersicon*) and the Rosidae, including *Vitis*, *Populus *and *Arabidopsis *[29]. Given the very incomplete view of the *Amborella *genome that is available in the BES data, we are not able to assess synteny between *Amborella *FPC contigs. Nevertheless, comparisons between the *Amborella *contigs and sets of syntenic blocks in the *Vitis *genome indicate that the γ triplication most likely occurred sometime after the divergence of all other angiosperms from the lineage leading to *Amborella*.

All BESs were compared to all annotated protein-coding genes in the *Vitis *genome placed within the context of the pre-triplication ancestral gene blocks and post-triplication syntenic segments identified by Tang *et al*. [29]. A total of 328 *Amborella *FPC contigs had between two and eight genes with significant best BLASTX matches (e-values ≤1.0E-6) to *Vitis *genes corresponding to pre-triplication gene blocks in the ancestral genome. In most of these cases (199 of 328; Additional file 2), best hits were distributed between two or three homeologous (that is, post-triplication) syntenic *Vitis *genome segments. Of the remaining 129 *Amborella *FPC contigs with BESs showing significant BLASTX hits to a single *Vitis *subgenome (that is, single copy of a triplicated ancestral block), most (113) included just 2 genes mapping to the ancestral *Vitis *gene blocks (14 including 3 genes, and 2 including 4 genes) (Additional file 2). All 21 FPC contigs with best BLASTX matches to five or more genes within the ancestral *Vitis *blocks were distributed among two or three post-triplication subgenomes. Complete sequences for the *Amborella *BAC contigs may reveal more even distribution of segments among *Vitis *subgenomes, but the results described here suggest that triplication, fractionation and divergence of homeologous segments in the *Vitis *genome postdate the divergence between lineages leading to *Vitis *and *Amborella *(that is, the last common ancestor of all extant angiosperms).

### Analysis of complete sequences for two *Amborella *BAC contigs

**Figure 4:**
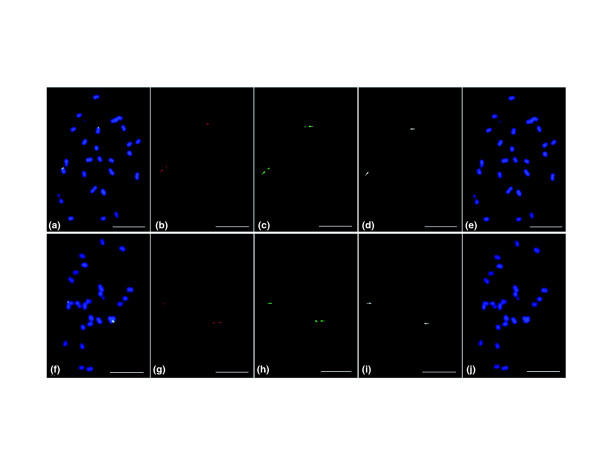
**Hybridization of three BAC clones in the minimum tiling paths for contigs 1003 and 431 to mitotic squashes (2*n *= 26) verifies the FPC assemblies. (a-e) **Results for contig 1003; **(f-j) **results for contig 431. Panels (a) and (f) show all three BAC-FISH probes merged; (e,j) DAPI staining; (b,c,d) show each of three BACs (red, green, white) for contig 1003; (g,h,i) show each of three BACs (red, green, white) for contig 431.

Two of the larger (approximately 500 kb) BAC contigs (IDs 431 and 1003) mapping to multiple segments in all four sequenced reference genomes were identified for further investigation. A minimum tiling path was constructed for each contig, and florescence *in situ *hybridizations were performed to verify that the BACs mapped to a single contiguous region in the *Amborella *genome (Figure 4). Each BAC in the tiling paths was subcloned and sequenced to 8 × coverage on an ABI 3730xl sequencer. Gaps were closed for each scaffold, and contiguous 487,318 and 629,678 bp phase II sequences were assembled for contigs 431 and 1003, respectively.

The DAWGPAWS suite of scripts was used to organize *ab initio *gene predictions, BLAST results and the output of repeat identification tools [61,62]. *Ab initio *gene predictions were generated using FGENESH [63], AUGUSTUS [64], SNAP [65], GeneID [66] and GenScan [67]. In addition, *Amborella *EST sequences produced by the 454 Titanium platform (2,943,273 reads; total read size of approximately 776 Mbp; average read length of 263.60 bp) and Sanger sequencing (38,147 reads; total read size of approximately 21.3 Mbp; average read length of 559.57 bp) were splice-aligned to the contigs using GMAP (Genomic Mapping and Alignment Program) [68] with the PASA (Program to Assemble Spliced Alignments) genome annotation tool [69]. All predictions were manually compared with BLASTX results against gene annotations from *Arabidopsis *[70], *Vitis *[45], *Z. mays *[56], *Medicago *[71], *Oryza *[72,73], and *Sorghum *[55] as well as tBLASTx results against the *Amborella *transcript assemblies. GBrowse views of gene annotations and BLAST results for each contig are available at the Ancestral Angiosperm Genome Project website [25].

**Figure 5:**
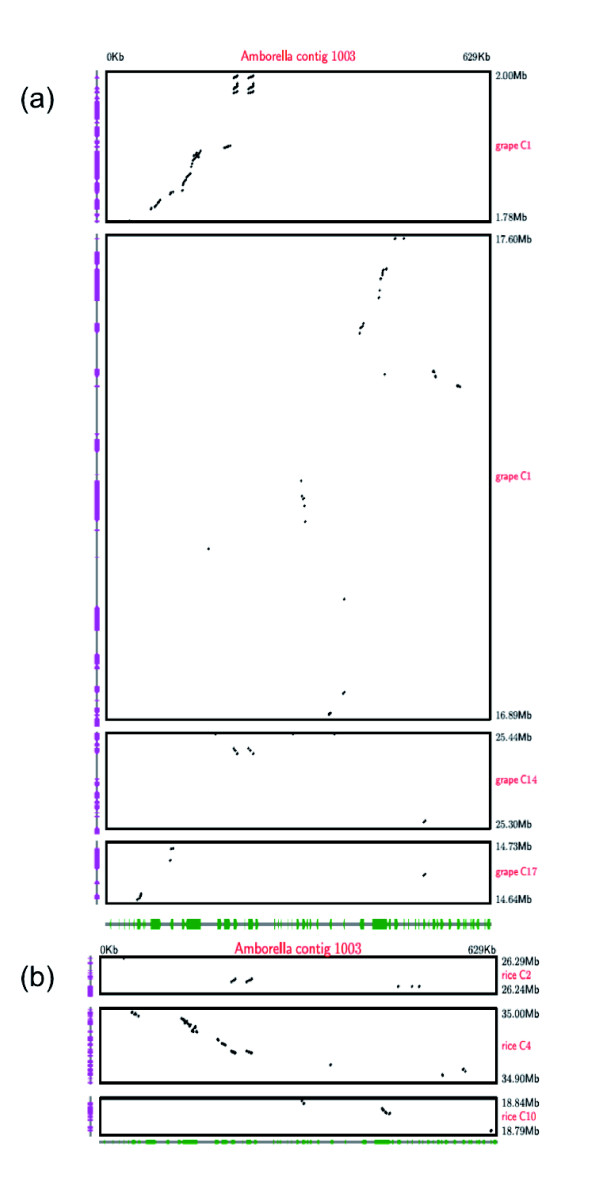
**LASTZ dot plots comparing BAC contig 1003 syntenic regions in the grape and rice genomes**. **(a) **Grape genome; **(b) **rice genome.

**Figure 6:**
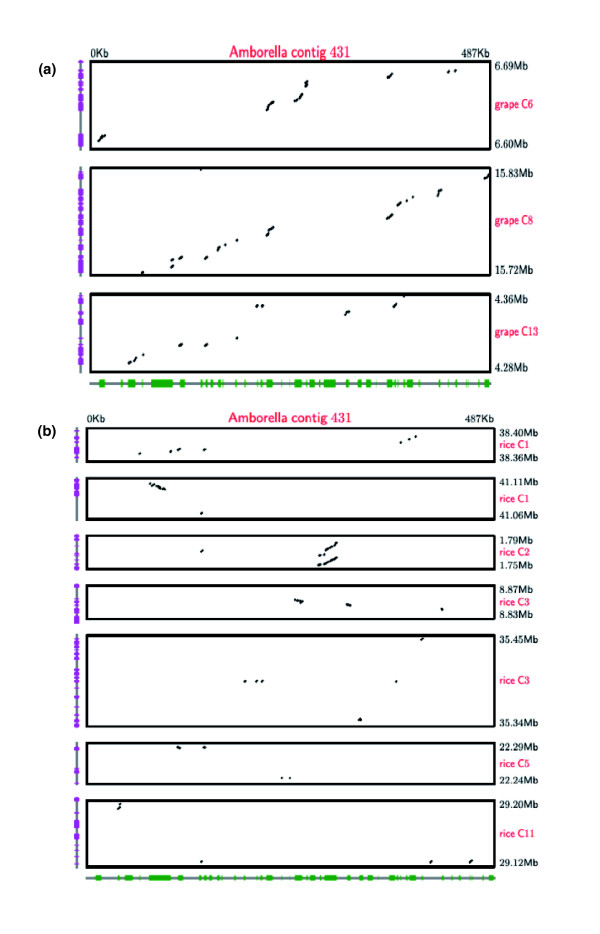
**LASTZ dot plots comparing BAC contig 431 syntenic regions in the grape and rice genomes**. **(a) **Grape genome; **(b) **rice genome.

Rigorous assessments of synteny between these *Amborella *contigs and the aforementioned four angiosperm genomes were performed using LASTZ [74,75]. Dotplots comparing the *Amborella *contigs and the *Vitis *genome show that contigs are syntenic with previously triplicated blocks [29]. Regions of contig 1003 match genes on syntenic segments of chromosomes 1, 14 and 17 in the *Vitis *genome (Figure 5) and contig 431 mapped to syntenic portions of *Vitis *chromosomes 6, 8 and 13 (Figure 6). These findings support the conclusion from the BES analyses suggesting that the γ triplication occurred after the first branching event in the phylogeny of extant angiosperms.

At least two genome duplications (ρ and σ) have been inferred to have occurred within the monocot lineage leading to rice since divergence of monocots and eudicots [28]. These duplications were evident in comparisons with both *Amborella *contigs. Regions of contig 1003 were found to be syntenic with portions of rice chromosomes 2 and 4 derived from the ρ duplication and a portion of chromosome 10 (Figure 5) that is related to these two regions through the earlier σ duplication [28]. The LASTZ analysis of contig 431 revealed synteny with seven regions in the rice genome (Figure 6) and one of the 'putative ancestral regions' (PAR 17) characterized by Tang *et al*. [28]. These PARs were defined as regions of synteny between the rice and *Vitis *genomes. Phylogenetic analyses of genes in *Amborella *contig 431 and syntenic regions of the rice and *Vitis *genomes may elucidate the timing of the γ triplication and genome duplications evident in synteny analyses of the rice genome relative to the divergence of monocots and eudicots.

### Phylogenetic analyses of gene families represented in sequenced *Amborella *contigs

While the fractionation process has resulted in the loss of most duplicated genes following the ancient polyploidy events evident in the syntenic *Vitis *and rice segments shown in Figures 5 and 6, duplicate *Vitis *genes have been retained for homologs of three *Amborella *genes located on contig 431 (Figures 6a). These genes were used to search the PlantTribes gene family database [35]. The three gene sets identified in the synteny analysis correspond to three gene families (auxin-independent growth promoter, ceramidase and plant uncoupling mitochondrial protein) circumscribed through OrthoMCL clustering [76] of gene annotations from the available *Arabidopsis*, *Carica *(papaya), *Populus*, *Medicago *(alfalfa), *Glycine*, *Cucumis *(cucumber), *Vitis*, *Mimulus*, *Oryza*, *Sorghum*, *Selaginella *(spike moss) and *Physcomitrella *genomes. Homologous genes sampled from exemplar asterid, ranunculid, non-grass monocot and gymnosperm species were obtained from EST assembly databases [25,77,78] and were added to each gene family set. Sequences in each gene family set were aligned using MUSCLE [79], and RAxML [80] run with the GTRGAMMA substitution model was used to obtain maximum likelihood estimates of gene trees.

**Figure 7:**
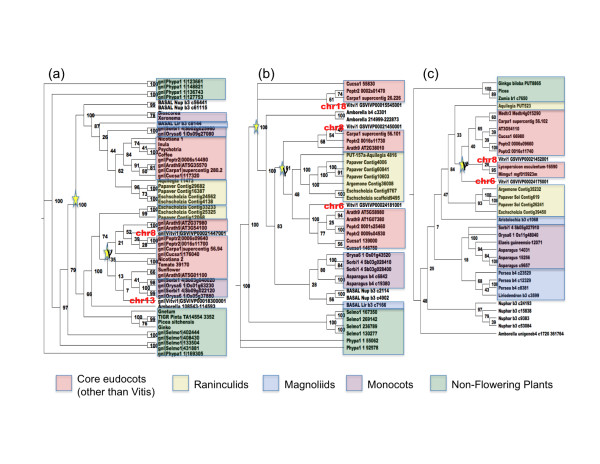
**Gene trees for auxin-independent growth promoter (*AXI1*), ceramidase and plant uncoupling mitochondrial protein 1 (*PUMP1*) gene families. (a) **Auxin-independent growth promoter (*AXI1*); **(b) **ceramidase; **(c) **plant uncoupling mitochondrial protein 1 [*PUMP1*] gene families. The gene trees show divergence of genes on *Amborella *contig 431 diverging from lineages leading to *Vitis *γ homeologs mapping to syntenic blocks on chromosomes 6, 8 and 13 (shown in red). Genes sampled from major angiosperm lineages are highlighted.

Inspection of the resulting gene trees shows support for the inference drawn from the BAC end sequence analysis. The γ triplication (hexaploidy event) clearly occurred after *Amborella *diverged from other extant angiosperm lineages (Figure 7). The placement of the γ triplication with respect to the divergence of monocots and eudocots or core eudicots and the Ranunculales varies among the three gene trees. This incongruence among gene trees is likely due to artifacts associated with substitution rate variation and insufficient taxon sampling. Analyses of additional gene families with broader taxon sampling will be necessary to obtain better resolution for the timing of the γ triplication with respect to the divergence of monocot, eudicots, Ranunculales (that is, 'basal' eudicots) and core eudocots.

## Conclusions

*A. trichopoda *is the sister species to the large clade encompassing all other extant flowering plants. As such, comparative analyses of *Amborella *and other flowering plants offer a uniquely informative perspective on the most recent common ancestor of all extant angiosperms. The physical map and BAC end sequences described in this study provide a low-resolution view of the *Amborella *genome. Nonetheless, these data shed light on genomic features of the last common ancestor of flowering plants. Moreover, the *Amborella *genome provides a unique reference for understanding genome evolution throughout angiosperm history. When placed in the context of the physical map, BESs representing just 5.4% of the *Amborella *genome allowed reconstruction of ancestral gene blocks in regions represented by 29 BAC contigs and inference of the timing of structural mutations that disrupted these blocks (Figure 3).

Analyses of BESs and BAC contigs also indicate that the ancient γ polyploidy event inferred from the *Arabidopsis *[58], *Carica *[81], *Populus *[60], and *Vitis *[45] genomes occurred after the *Amborella *lineage diverged from the rest of the angiosperms. Therefore, if the origin of angiosperms was associated with a genome duplication as has been hypothesized elsewhere [16,20,23], that polyploidy event predated the γ event.

## Materials and methods

### BAC library construction

Protocols for DNA megabase preparation, library construction, picking and arraying proposed in Luo and Wing [82] were followed.

### Fingerprinting

The SNaPshot fingerprinting technique was adopted [32] with the modifications described by Kim *et al*. [83]. Snapshot reactions were loaded into ABI 3730xl DNA sequencers. Analysis of data for each contig was carried out using the ABI Data Collection Program.

### Physical map construction

Fingerprints were assembled into contigs using the program FPC version 7.2 [34]. The initial assembly was carried out using a Sulston score threshold of e-50 followed by three rounds of dequeuing at the same stringency and auto-merging of contigs at e-21.

### BAC end extraction and sequencing

BAC DNA was extracted and end sequenced from 36,684 clones using the methods described by Ammiraju *et al*. [83,84]. Sequence quality assessment and trimming were carried out using the programs Phred [85] and Lucy [86].

### Random sheared library

A random sheared library was constructed as previously described [87].

### cDNA sequencing and assembly

Additional Sanger ESTs were generated from available male and female flower bud cDNA libraries [10] (Table 4). Libraries for 454 sequencing were constructed from the tissues listed in Table 4 using the Mint cDNA synthesis kit (Evrogen, Moscow, Russia). Total RNAs for cDNA synthesis were isolated using a combination of CTAB extraction and the RNeasy Plant Mini kit (Qiagen Valencia, CA USA) as previously described for basal angiosperms [11]. Two rounds of messenger RNA isolation were performed with the Poly(A)Purist™ mRNA Purification Kit (Ambion Inc. Austin, TX USA) according to the manufacturer's recommendation. Contaminant DNA was removed with DNA-free™ (Ambion Inc.) and mRNA quality was verified using a Bioanalyzer (Agilent Inc. Santa Clara, CA, United States). Vector and adaptor sequences were trimmed from 454 Titanium (2,943,273 reads; total read size of approximately 776 Mbp; average read length of 263.60 bp) and Sanger sequences (38,147 reads; total read size of approximately 21.3 Mbp; average read length of 559.57 bp) using seqclean [88] and assembled using MIRA [89].

### Similarity searches, repeat classification and contig anchoring

Similarity searches were carried out using the programs BLASTN and BLASTX [39]. BLASTN was run under relaxed settings (-q -4 -r 5) in order to accommodate the evolutionary distance between *Amborella *and the species included in the repeat databases used; the significance threshold was set at 1e-10. In the case of BLASTX searches the threshold was set at 1e-5 or 1e-4 for the BES synteny analysis. tBLASTX was used to anchor the contigs to the reference genomes (see Results for details).

### Databases

The databases used in similarity searches were RepBase version 15.08 [38], the GenBank non-redundant (nr) database, and the *Oryza*, *Arabidopsis*, *Vitis *and *Populus *genome sequences.

### Validation of repeat searches and MITE identification

The program MUST [49] was used for *de novo *characterization of highly repeated sequences; results were then inspected for the presence of MITE features. Inverted repeats were identified manually parsing the results of dot-plot comparisons made using the program 'Dotter' [90].

### Simple sequence repeat searches

Microsatellites were identified using the program Sputnik [91]. SSR composition, length and distribution were parsed and analyzed using the tools and the strategy used by Morgante *et al*. [92].

### Fluorescence *in situ *hybridization

FPC contigs were validated by hybridizing BAC DNAs to *Amborella *chromosome squashes. DNA was prepared for BAC mapping to the middle and both ends of BAC contigs 431 and 1003 and used to prepare fluorescently labeled BAC-FISH probes. Chromosome squashes were prepared from root tips and labeled BAC-FISH probes were prepared as described by Xiong *et al*. [93].

### Contig sequencing and annotation

Minimum tiling paths of seven and six BACs were identified for contigs 1003 and 431, respectively, by the visual inspection of the FPC assemblies. Adjacent clones were chosen based on their reciprocal position and probability value associated to their overlapping fingerprinted bands as shown by FPC. Sequencing of selected minimum tiling path BACs was done to phase II quality as previously described [73]. Phase II BAC sequences were then assembled into 1003 and 431 contig sequences based on dot plot comparisons and overlap similarity between adjacent clones.

Perl scripts available from the DAWGPAWS package [61,62] were used to convert computational annotation results from multiple sources into a single GFF3 file for combined evidence annotation in Apollo [94] and publication in Gbrowse [95]. *Ab initio *gene annotation programs used in this process included FGENESH [63] AUGUSTUS [64], SNAP [65], GeneID [66] and GenScan [67]. Because *Amborella*-specific gene model parameterizations were not available for these programs, multiple plant models were used for each *ab initio *program. The sequence of the entire contig was BLASTx (e < 1 × 10^-5^) searched against gene annotations from *Arabidopsis *[70], *Vitis *[45], *Z. mays *[56], *Medicago *[71], *Oryza *[72], and *Sorghum *[55] as well as tBLASTx (e < 1 × 10^-5^) searched against a database of comprehensive *Amborella *transcript assemblies [25]. In addition, *Amborella *EST sequences (reads and assemblies; Table 4) were splice-aligned to the contigs using GMAP (Genomic Mapping and Alignment Program) [68] with the PASA (Program to Assemble Spliced Alignments) genome annotation tool [69]. The gene models and BLAST search results were manually combined into gene models using the Apollo genome annotation curation tool [94].

### Synteny analysis of sequenced BAC contigs with *Vitis *and *Oryza *genomes

Sequenced *Amborella *BAC contigs 431 (487,318 bp) and 1003 (629,678 bp) were compared to the International Rice Genome Sequencing Project (IRGSP) rice genome assembly (version 5) and the Genoscope 12 × *Vitis *genome assembly using LASTZ and default parameters. Prior to LASTZ comparisons, all genomic sequences were masked using NCBI's WindowMasker to remove simple repeats. Significant matches after repeat masking were visualized as dot plots. Gene annotations for the rice and *Vitis *genomes were obtained from the Rice Annotation Project [96] and Genoscope [97], respectively, and plotted on the vertical axes of the dot plots (Figures 5 and 6). FGENESH [63] annotations for the *Amborella *contigs were included on the horizontal axes of the dot plots. LASTZ scores were summed for all aligned *Amborella*-rice or *Amborella-Vitis *blocks within 100 kb of each other in sequenced genomes. All regions with summed scores >100,000 were considered as syntenic and included in Figures 5 and 6.

### Phylogenetic analysis

All alignments were carried out using the program 'MUSCLE' [79] run under default settings. Maximum likelihood analyses were run on aligned DNA and amino acid sequences using RAxML [80] and the GTRGAMMA nucleotide substitution model.

### Submission of data to GenBank databases

BESs (HR616970 to HR686434), full-length BAC sequences (AC243594.1 to AC243606.1), Sanger shotgun sequences (HR614237 to HR616931), 454 shotgun sequences (SRP006044), Sanger ESTs (FD425831.1 to FD443502.1) and 454 cDNA sequences (SRX018174, SRX018165, SRX018164, SRX018163, SRX018157, SRX018156) have been deposited in the appropriate NCBI GenBank sequence databases. All sequences are also available at the Ancestral Angiosperm Genome Project website [25].

## Abbreviations

bp: base pair; BAC: bacterial artificial chromosome; BES: BAC end sequence; EST: expressed sequence tag; FISH: fluorescence *in situ *hybridization; HICF: high information content fingerprinting; LINE: long interspersed element; LTR: long terminal repeat; MITE: miniature inverted repeat transposable element; SSR: simple sequence repeat; TE: transposable element.

## Authors' contributions

JLM, AZ, RAW and CWD designed and coordinated the study. The *Amborella *BAC library was constructed and characterized in the Arizona Genomics Institute (AGI) by DK, YY, KC, JLG, AS and ML. cDNA library production and sequencing was performed by AC at the University of Florida and assemblies were performed by SA. Funding for BAC library construction was obtained by DM, JB, JEC, JT, CWD and RAW. Comparative analyses were performed by AZ, AS, JEB, JCE, JD, HT, SR, AHP, DES, PSS, VAA, HM, CWD and JL-M. Florescence *in situ *hybridizations were performed by ZX and JCP. BAC contig annotations were performed by JEB, JCE, SC, BB and JLM. AZ and JLM wrote the first draft of the manuscript and all authors contributed to refinement.

## Supplementary Material

Additional file 1 **Supplemental tables and figures cited with additional details for the physical map and shotgun sequences**. Click here for file

Additional file 2 **Synteny analysis of *Amborella *BAC ends and *Vitis *genes**. Click here for file
