# Supplementary material for: A physical map for the Amborella trichopoda genome sheds light on the evolution of angiosperm genome structure
Source: Genome Biol. 2011 May 27;12(5):R48. doi: 10.1186/gb-2011-12-5-r48 (PMC3219971; doi:10.1186/gb-2011-12-5-r48)
Supplement: Additional file 1 — Supplemental tables and figures cited with additional details for the physical map and shotgun sequences. [file gb-2011-12-5-r48-S1.DOC]

Additional file 1:

**Figure S1**. FPC maps for BAC contigs including verified positive probes for Amborella homologs of a) *ASD* (At1g14810), b) *DWARF1* (At3g19820), c) *GIGANTEA* (At1g22770), d) *LEAFY* (At5g61850), e) dienelactone hydrolase (At2g32520), f) cytochrome-C-oxidase related gene (At4g37830), g) *EIF3K* (At4g33250) and h) a hypothetical protein-coding gene with strong similarity to rice gene Os02g0593400 (At5g63135).

**
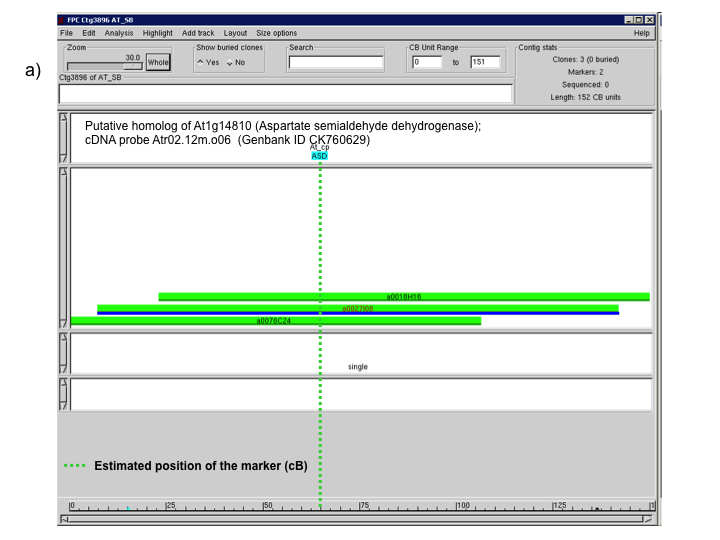
**

**
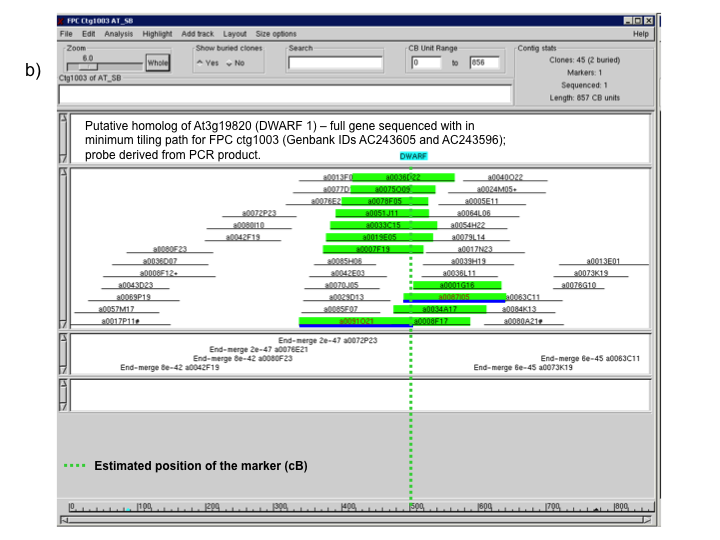
**

**
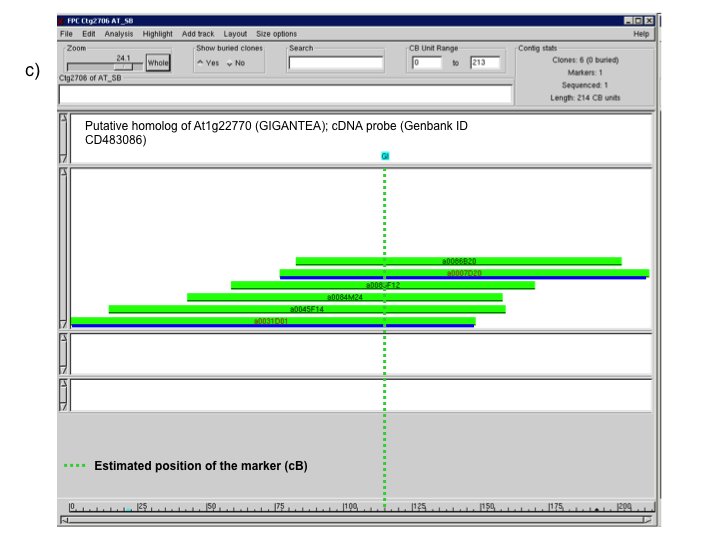
**

**
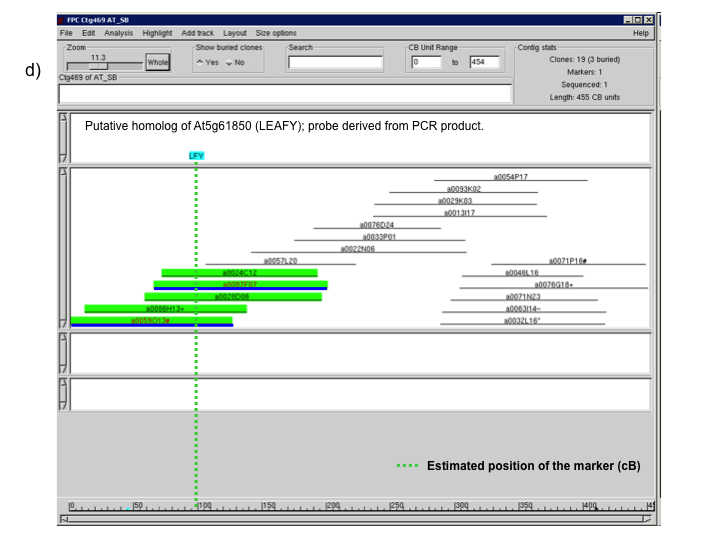
**

**
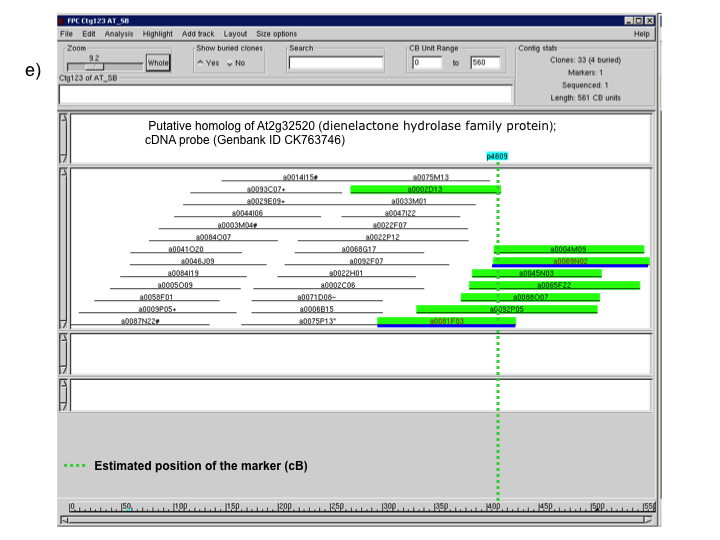
**

**
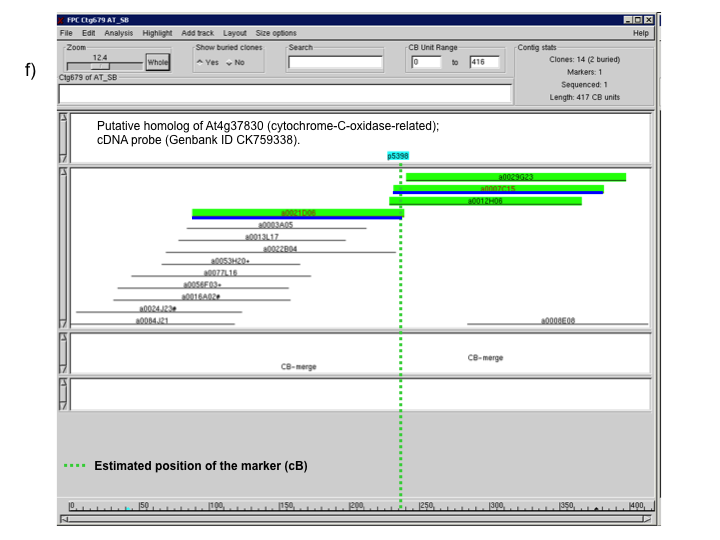
**

**
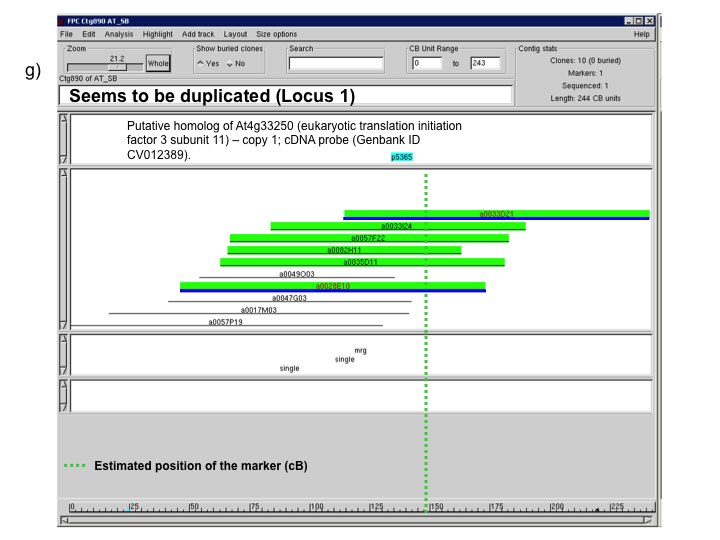
**

**
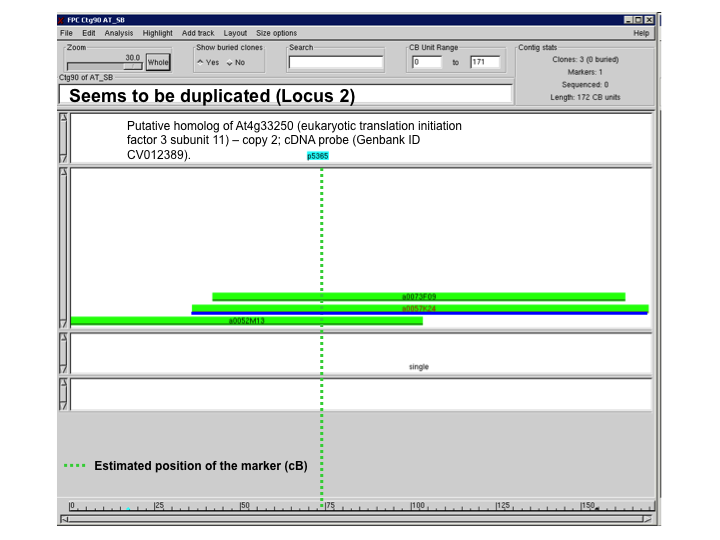
**

**
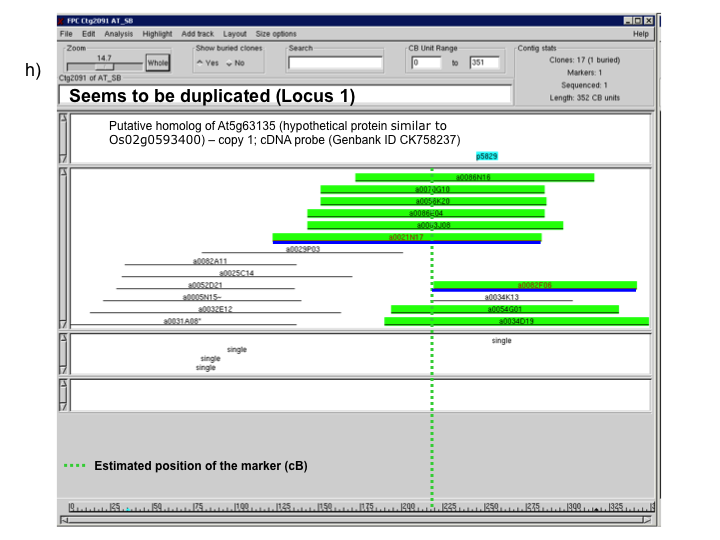
**

**
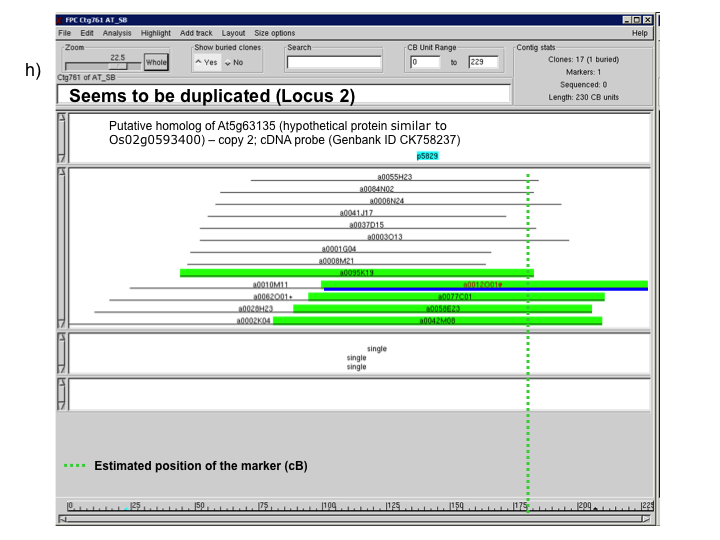
**

**Figure S2**. A plot of BAC number vs. HICF bands for each FPC contig shows three BAC contigs departing from an otherwise linear relationship.


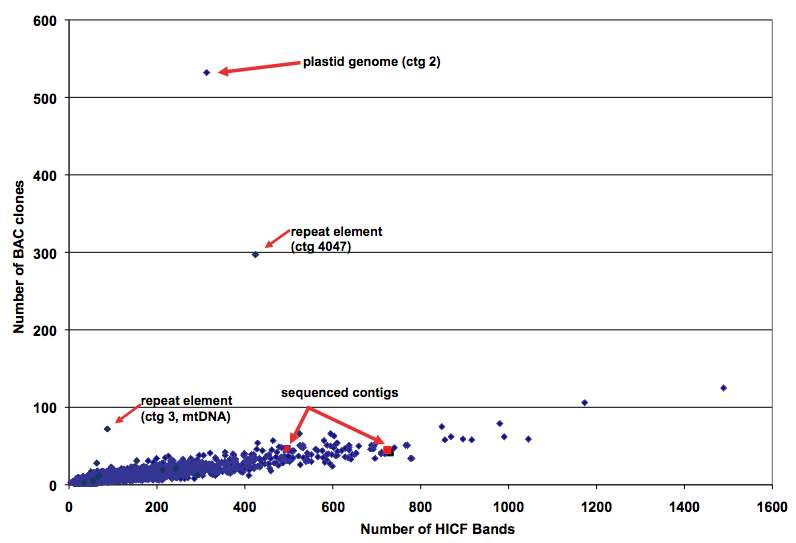


**Figure S3.** Sequences for putatively high copy MITES identified in the BES and SGS data. Terminal inverted repeat (TIR) and target site duplication (TSD) sequences are highlighted.

Table S1: Distribution of TE types in 648,519 454 survey sequences shows frequencies similar to those observed in the Sanger shotgun and BAC end sequences (Table 1). Values in parentheses include matches found in comparisons of initially unclassified sequences and those that had been classified in Repbase search (I; see text.)

|  | **Type** | **Absolute number in BESs** | **% BESs** | **% Repeats in BESs** | **Absolute number in SGSs** | **% SGSs** | **% Repeats in SGSs** | **Absolute number in 454 reads** | **% 454 Reads** |
| --- | --- | --- | --- | --- | --- | --- | --- | --- | --- |
| **DNA-TEs** | hAT | 642 (1671) | 0.92 (2.41) | 6.84 (4.61) | 20 (41) | 0.74 (1.52) | 5.73 (2.94) | 4076 | 0.63 |
|  | MuDR | 343 (724) | 0.49 (1.04) | 3.65 (2.00) | 7 (30) | 0.26 (1.11) | 2.00 (2.15) | 1485 | 0.23 |
|  | CACTA | 27 (75) | 0.04 (0.11) | 0.29 (0.21) | 0 (4) | 0 (0.15) | 0 (0.29) | 12 | 0.00 |
|  | Helitrons | 12 (69) | 0.02 (0.10) | 0.13 (0.19) | 0 (3) | 0 (0.11) | 0 (0.22) | 326 | 0.05 |
|  | Other | 108 (595) | 0.15 (0.86) | 1.15 (1.64) | 1 (24) | 0.04 (0.89) | 0.29 (1.72) | 1816 | 0.28 |
|  | **Total** | 1132 (3134) | 1.63 (4.51) | 12.06 (8.64) | 28 (102) | 1.04 (3.78) | 8.02 (7.31) | 7715 | 1.19 |
| **Retrotransposons** | LTR Ty1-*copia* | 2162 (9578) | 3.11 (13.79) | 23.02 (26.42) | 64 (314) | 2.37 (11.65) | 18.34 (22.51) | 15275 | 2.36 |
|  | LTR Ty3-*gypsy* | 2431 (8395) | 3.50 (12.09) | 25.89 (23.15) | 129 (377) | 4.78 (13.98) | 36.96 (27.03) | 29583 | 4.56 |
|  | LTR not classified | 720 (2868) | 1.04 (4.13) | 7.67 (7.91) | 51 (139) | 1.89 (5.16) | 14.61 (0.96) | 6525 | 1.01 |
|  | LINEs | 1876 (8055) | 2.70 (11.60) | 19.98 (22.22) | 55 (294) | 2.04 (10.91) | 15.76 (21.08) | 16053 | 2.48 |
|  | SINEs | 11 (183) | 0.02 (0.26) | 0.12 (0.50) | 0 (4) | 0 (0.15) | 0 (0.29) | 567 | 0.09 |
|  | Retro not classified | 1058 (4046) | 1.52 (5.82) | 11.27 (11.16) | 23 (165) | 0.85 (6.12) | 6.59 (11.83) | 218 | 0.03 |
|  | **Total** | 8258 (33125) | 11.89 (47.69) | 87.94 (91.36) | 321 (1293) | 11.91 (47.96) | 91.98 (92.69) | 68221 | 10.52 |
|  |  |  |  |  |  |  |  |  |  |
| **Total** |  | 9390 (36259) | 13.52 (52.20) | 100 (100) | 349 (1395) | 12.95 (51.74) | 100 (100) | 75936 | 11.71 |

Table S2. Identity of FPC contigs anchored to at least one region of one of the four sequenced reference genomes. Contigs anchored to more than one regions in a genome show more than one “region hit”. Contigs were considered anchored if they had at least four positive hits (e-value lower than 1e-4) to at least 3 distinct genes (see text). The number of BES matching *Amborella* cDNA sequences (Table 4) is also shown.

| **ALL** | **BES (non-repetative)** | **BES matching Amborella cDNAs** | **Arabidopsis Anchoring** | **A (regions hit)** | **Oryza Anchoring** | **O (regions hit)** | **Poplar Anchoring** | **P (regions hit)** | **Vitis anchoring** | **V(regions hit)** |
| --- | --- | --- | --- | --- | --- | --- | --- | --- | --- | --- |
| Cntg51 | 56 | 32 | NO | 0 | NO | 0 | * | 1 | * | 1 |
| Cntg 53 | 30 | 17 | NO | 0 | * | 1 | * | 1 | * | 1 |
| Cntg1003 | 52 | 32 | * | 3 | * | 4 | * | 3 | * | 3 |
| Cntg104 | 46 | 31 | NO | 0 | NO | 0 | * | 1 | NO |  |
| Cntg133 | 20 | 14 | * | 1 | NO | 0 | * | 1 | * | 1 |
| Cntg134 | 30 | 21 | NO | 0 | NO | 0 | * | 1 | * | 1 |
| Cntg140 | 35 | 28 | * | 1 | * | 1 | * | 1 | * | 1 |
| Cntg162 | 71 | 42 | NO | 0 | * | 1 | * | 1 | * | 1 |
| Cntg1790 | 39 | 28 | NO | 0 | NO | 0 | NO | 0 | * | 1 |
| Cntg278 | 26 | 20 | * | 1 | * | 1 | NO | 0 | NO | 0 |
| Cntg35 | 92 | 60 | NO | 0 | NO | 0 | NO | 0 | * | 1 |
| Cntg357 | 61 | 34 | * | 1 | * | 2 | * | 2 | * | 1 |
| Cntg423 | 57 | 34 | * | 1 | NO | 0 | NO | 0 | * | 2 |
| Cntg428 | 35 | 19 | * | 1 | NO | 0 | NO | 0 | * | 1 |
| Cntg431 | 56 | 41 | * | 4 | * | 4 | * | 4 | * | 3 |
| Cntg47 | 62 | 29 | * | 2 | * | 1 | * | 1 | * | 2 |
| Cntg676 | 38 | 24 | * | 1 | * | 1 | * | 1 | * | 2 |
| Cntg692 | 39 | 19 | NO | 0 | * | 1 | * | 1 | NO | 0 |
| Cntg77 | 47 | 30 | NO | 0 | * | 2 | * | 1 | NO | 0 |
| Cntg779 | 52 | 49 | * | 1 | NO | 0 | NO | 0 | * | 1 |
| Cntg78 | 48 | 24 | * | 1 | NO | 0 | * | 1 | * | 0 |
| Cntg866 | 55 | 40 | * | 2 | * | 1 | * | 2 | * | 2 |
| Cntg895 | 75 | 43 | * | 5 | * | 2 | * | 5 | * | 3 |
| Cntg9 | 74 | 49 | * | 2 | * | 1 | * | 3 | * | 2 |
| Cntg179 | 118 | 114 | * | 5 | * | 3 | NO | 3 | * | 3 |
| Cntg44 | 32 | 19 | NO | 0 | NO | 0 | NO | 0 | * | 1 |
| Cntg198 | 45 | 29 | NO | 0 | NO | 0 | * | 1 | * | 1 |
| Cntg415 | 34 | 22 | NO | 0 | NO | 0 | * | 1 | * | 1 |
| Cntg122 | 54 | 32 | NO | 0 | NO | 0 | * | 1 | * | 1 |

* indicates matches to genes in syntenic regions
